# Preliminary Evidence of *Rickettsia slovaca* and *Rickettsia conorii* Infection in the Sera of Sheep, Dogs and Deer from an Area of Northern Spain

**DOI:** 10.3390/pathogens10070836

**Published:** 2021-07-03

**Authors:** Lourdes Lledó, Consuelo Giménez-Pardo

**Affiliations:** Departamento de Biomedicina y Biotecnología, Universidad de Alcalá, Ctra. Madrid-Barcelona km 33.6, 28805 Alcalá de Henares, Spain; consuelo.gimenez@uah.es

**Keywords:** epidemiology, *Rickettsia slovaca*, *Rickettsia conorii*, sheep, dog, deer

## Abstract

Limited information is available on the presence of rickettsial infection in animal reservoirs in Spain. Antibodies against *Rickettsia slovaca* and *Rickettsia conorii* were therefore sought in the sera of farm, domestic and wild animals (*n* = 223 samples) in an area of northern Spain. Indirect immunofluorescence assays showed: (A) 17/120 and 16/120 (14.2% and 13.3%) of serum samples from sheep (farm animals) reacted with *R. slovaca* and *R. conorii* antigens, respectively; (B) 10/73 and 10/73 (13.7% and 13.7%) of samples from dogs (domestic animals) did the same; (C) as did 22/30 and 20/30 (73.3% and 66.6%) of samples from deer (wild animals) (overall titre range: 1/40 to 1/1280). The prevalence of both types of infection was significantly greater in the wild animals than either the farm or domestic animals. The largest titres were recorded for *R. slovaca* in all three groups.

## 1. Introduction

Rickettsia are obligate intracellular, Gram-negative bacteria. They are transmitted by ticks, both transovarially, i.e., without the need of a vertebrate reservoir, and trans-stadially, i.e., between tick life stages, but requiring a mammalian reservoir [1]. However, recent studies from Central Europe have suggested the role of transovarial transmission to be limited [2]. 

*Rickettsia slovaca* is the aetiological agent of tick-borne lymphadenopathy. The tick *Dermacentor marginatus* is the main vector of *R. slovaca* in the Mediterranean Basin (southern Europe and North Africa) [3,4]. *Rickettsia conorii* causes the zoonotic disease Mediterranean spotted fever, which is transmitted by *Rhipicephalus sanguineus* sensu lato and appears to be endemic throughout Spain [5]. Both *R. slovaca* and *R. conorii* have an important impact on public health.

In Slovakia, the prevalence of *R. slovaca* in adult *D. marginatus* ticks collected from sheep and vegetation was reported as 3% and 27%, respectively [6]. In southern Croatia, Punda-Pollic et al. [7] used molecular detection techniques to show that *D. marginatus* ticks collected from domestic animals were infected with *R. slovaca*. In Romania, PCR analysis showed adult ticks from sheep, goats and horses to carry the same bacterium [8]. Chisu et al. [9] detected *R. slovaca* in several species of tick collected from vegetation, foxes, swine, wild boar and mouflon in Sardinia (Italy). In Corsica (France), Cicculli et al. [10] detected *R. slovaca* in *D. marginatus* from wild boar. In Greece (where spotted fever group (SFG) rickettsial disease is endemic), *R. slovaca* was found in ticks collected from sheep [11]. In northeastern Spain, Ortuño et al. [12] recorded indirect immunofluorescence assay (IFA)-determined seroprevalences for *R. slovaca* of 15.7% in sheep, 20.8% in goats and 65% in bullfighting cattle. Using real-time PCR, the same authors detected evidence of *R. slovaca* infection in goat blood. Antibodies to *R. conorii* are commonly detected in dogs in northern Spain; Solano-Gallego et al. [13] recorded a prevalence of 56.4% in northeastern Spain and of 24.6% in the Spanish northwest [14]. 

The aim of the present work was to extend our epidemiological knowledge of rickettsial infections in northern Spain. The objective of the work was to detect the seroprevalence of antibodies against the group of spotted fevers in various animal species that have contact with humans using various species of this group of rickettsias as antigens to increase the sensitivity of the study. The choice of antigens to be used was made among those species of rickettsias of the group of spotted fevers that on occasion have been detected in the country (Spain) and in the animals to be studied. Evidence of infection by *R. slovaca* (an emerging pathogen in ruminants) and *R. conorii* (a prevalent pathogen in domestic and wild animals) was therefore sought in the sera of sheep, dogs and deer via IFAs.

## 2. Results

### 2.1. Sheep

All animals had ticks and fleas, but they were not collected. Seventeen sheep had antibodies to *R. slovaca* (seroprevalence: 14.2%; 17/120). Sixteen of these very same animals had antibodies to both pathogens (seroprevalence: 13.3% each; 16/120). Seropositive animals (i.e., for either pathogen) were detected in all four herds (Flock 1, 16.6% [5/30]; Flock 2, 30% [9/30]; Flock 3, 3.3% [1/30]; Flock 4, 6.6% [2/30]). However, in Flocks 1 and 2, the combined seroprevalence of *R. slovaca* and *R. conorii* (23.3% [14/60]) was greater than in Flocks 3 and 4 (5% [3/60]) (*χ^2^* = 8.292, *p* = 0.003), despite them all being geographically quite close to one another. Combined seroprevalence by age was: >4 years 18.7% (9/48), 4–5 years 13.3% (8/60) and <5 years 0.00% (0/12) (*χ^2^* = 1.382, *p* = 0.239). Titres for samples seropositive to *R. slovaca* ranged between 1/40 and 1/1280 and to *R. conorii* between 1/40 and 1/320 (Table 1). 

### 2.2. Dogs

Ticks were found on 14 dogs: *Ixodes canisuga* (*n* = 13 ticks), *Ixodes hexagonus* (15), *Ixodes ricinus* (16), *Rhipicephalus sanguineus* (10), *Rhipicephalus pusillus* (1) and *Dermacentor reticulatus* (1). A total of 10 dogs (10/73: 13.7%) had antibodies to both *R. slovaca* and *R. conorii* (the remaining dogs were all seronegative for both pathogens). The gender distribution of the seropositive dogs was seven males (16.3% [7/43]) and two females (6.7% [2/30]) (*χ^2^* = 1.510, *p* = 0.219). Seroprevalence according to age was 4.5% (1/22) in dogs <4 years old, 11.1% (4/36) in those 4–7 years old and 33.3% (5/15) in >8 years old (*χ^2^* = 6.654, *p* = 0.035).

Antibodies to rickettsia were detected in comparable numbers across the dogs used for hunting (6/35 = 17.1%), as pets (3/29 = 10.3%) and for sheep-herding (1/3 = 33.3%) (*χ^2^* = 1.415, *p* = 0.492). Five dogs living with other animals had antibodies (5/41 = 12.2%), as did five dogs that lived without another animals (5/32 = 15.6%) (*χ^2^*= 0.178, *p* = 0.672). No significant difference in seroprevalence was seen between dogs that had ticks (4/14 = 28.5%) and those that did not have ticks (6/59 = 10.2%) (*χ^2^* = 3.241, *p* = 0.071). The titres for the seropositive samples to *R. slovaca* ranged from 1/80 to 1/1280, and for *R. conorii* from 1/40 and 1/1280 (Table 1).

### 2.3. Deer

All animals had ticks; a total of 93 were collected. The most common species identified were *Ixodes ricinus* (*n* = 85), followed by *Haemaphysalis punctate* (*n* = 5) and *Rhipicephalus bursa* (*n* = 3). Twenty two of the red deer had antibodies to *R. slovaca* (seroprevalence: 73.3%; 22/30), and twenty had antibodies to *R. conorii* (seroprevalence: 66.6%; 20/30) (*χ^2^* = 0.317, *p* = 0.573). Twenty animals had antibodies against both pathogens (seroprevalence: 66.6%; 20/30). The gender distribution of the seropositive deer was: for *R. slovaca*, 5 males (5/10 = 50%) and 17 females (17/20 = 85%) and for *R. conorii*, 5 males (5/10 = 50%) and 15 females (15/20 = 75%) (*χ^2^* = 1.875, *p* = 0.170). The titres of the samples seropositive for *R. slovaca* ranged from 1/80 to 1/1280 and for *R. conorii* from 1/40 to 1/640 (Table 1).

### 2.4. All Animal Groups

The red deer had the highest seroprevalence for both *R. slovaca* (*χ^2^* = 53.337, *p* < 0.00001) and *R. conorii* (*χ^2^* = 44.877, *p* < 0.00001). In all three groups of animals, the highest titres were recorded for *R. slovaca*.

## 3. Discussion

The present results revealed the presence of antibodies against SFG rickettsia in sheep, dogs and deer in the study area. While the interpretation of serological data is difficult given the cross-reactivities among SFG rickettsia when using IFA assays [15], serology is the easiest means of detecting them. In future work, other assays could be used to validate these IFA results. Therefore, the results were preliminary until they are confirmed with a more specific serological assay such as cross-adsorption, which will allow identifying with high specificity against which species of rickettsia these antibodies have been produced. However, we considered this to be a first step in the epidemiological study in the studied area and in the animals studied of the presence of infections of the group of SFG rickettsias and to provide some interesting data although the results were preliminary.

Ticks were present on at least some animals in every group studied; those on dogs and deer were identified, and of these identified ticks, several have been recognized as vectors of *R. slovaca* and *R. conorii*. Antibodies to *R. slovaca* and *R. conorii* were detected in members of all animal groups. In Croatia, *R. slovaca* has been detected in *Rh. sanguineus*, *D. marginatus* and *Hyalomma marginatum* ticks [7]. Leulmi et al. [16] detected *R. slovaca* in *Ha. punctate* ticks in Algeria, sometimes co-feeding with infected *D. marginatus*. In Spain, *D. marginatus* is the most important tick vector [17]. For *R. conorii*, *Rh. sanguineus* sensu lato is the main vector across the entire Mediterranean area, but other species of *Rhipicephalus* and *Ixodes* ticks may also act as vectors [5]. 

Little is known about the serological response to rickettsial infection in domestic ruminants (carriers of ticks that help maintain and transmit SFG rickettsia and that are often in contact with humans). This is the first time that antibodies against *R. slovaca* and *R. conorii* have been detected in sheep in this area of northern Spain. In these animals, the prevalence of *R. slovaca* antibodies was 14.2%, while for *R. conorii*, it was 13.3% (with higher titres for *R. slovaca*); prevalence was always higher in younger animals, as might be expected since in this region, older animals are taken out to pasture less often. The seroprevalence distribution (for both pathogens together) among the four sheep flocks was not homogenous. The higher seroprevalence seen in Flocks 1 and 2 suggested these animals may live under less hygienic conditions. Ortuño et al. [12] collected blood samples from sheep and goat in Catalonia (northeastern Spain) and determined the presence of *R. slovaca* by both serology (IFA) and molecular techniques. Antibodies were detected in 15/95 animals (15.8%). Two sheep showed antibody titres of ≥1/320. The titres reported in the present work were higher (up to 1/1280). The seroprevalence recorded for *R. conorii* across Spain can vary widely; this can be true even in the same region, e.g., 0% in Burgos [18] and 38.9% in Salamanca [19], both of which belong to Castilla y León.

Dogs are the main reservoir of *R. conorii*, and numerous studies have used them as sentinels [20]. Much less information exists for other SFG species such as *R. slovaca*. In the present work, the seroprevalence of both bacterial species was 13.6% in these animals, similar to the combined figure for sheep (13.3%). The antibody titres to *R. slovaca* were, however, higher. 

Rojo Vazquez [21] reported a seroprevalence for *R. conorii* in Castilla-León of 14.2% (greater in dogs used to guard sheep than those involved in hunting and security duties). In another study from the same region, Delgado and Cármenes [22] reported a prevalence of 23.4% (higher in animals with sheep-herding and guard duties or living in rural areas). The present results also highlighted a higher prevalence in sheepdogs (33.3%) and in old animals (33.3%), similar to that reported by Amusategui et al. [14]. The present work provided the first seroprevalence results for *R. slovaca* infection in dogs in Spain; the value was higher than that reported in Germany (2%) [23]. 

Rickettsial infections are described to have both a wild and domestic cycle, both involving ticks. The tick species associated with *R. slovaca* in ruminants are not completely known, although *D. marginatus* may play an important role [17]. *R. conorii* is associated with the tick *Rh. sanguineus* and can infest a wide range of wild animal host species [24]. In the present work, the highest seroprevalence to *R. slovaca* and *R. conorii* was detected in the wild deer studied (73.3% for *R. slovaca* and 66.6% for *R. conorii*). The highest antibody titres were always for *R. slovaca*, irrespective of animal species.

In summary, the present results expand our knowledge of the seroprevalence of SFG rickettsia in peridomestic and wild animals. The large number of seropositive animals detected (especially the wild animals studied) suggests that human contact with these animals is a risk factor for *R. slovaca* and *R. conorii* infection or other member of SFG rickettsia. Further research should attempt to determine the role of different species in the epidemiology and ecology of these infections and involve other areas of the country.

## 4. Materials and Methods

### 4.1. Study Area

**Figure 1:**
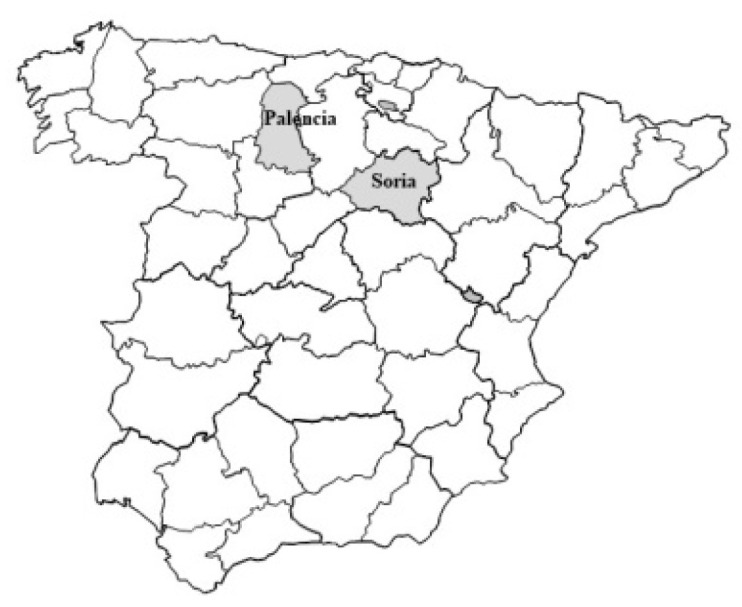
Palencia and Soria provinces.

This study was performed in the Spanish provinces of Palencia and Soria in the region of Castilla-León, which occupies the Northern Meseta of the Iberian Peninsula. The area is mainly rural with numerous isolated villages. The main activities are forestry, agriculture and cattle raising (Figure 1). The sera examined were collected over two years and stored at −20 °C until analysis.

### 4.2. Serum Samples and Tick Counts

#### 4.2.1. Sheep

Serum samples were collected from 120 animals belonging to four flocks. The age range of these animals was 2–12 years; the median age was 4 years (IQR 3–5 years). The median age of Flock 1 was 4.1 years, while that of Flock 2 was 3.7 years; the median age of these two flocks together (which came from the same locality) was 3.8 years. The median age of Flock 3 was 3.3 years, and that of Flock 4 was 4.9 years; the median age of these two flocks together (which came from the same locality, but different to that above) was 4.1 years. Blood was obtained from anaesthetized animals by jugular venipuncture. 

#### 4.2.2. Dogs

Serum samples were collected from 73 animals (43 males and 30 females) aged 1–14 years (22 were less than 4 years old; 36 were between 4 and 7 years old; 15 were over 8 years old; median age 5.4 years (IQR 4–7 years)). Thirty five animals were used for hunting, twenty-nine as pets, six as watchdogs, and three as sheepdogs. Thirty two of the sampled dogs lived with other dogs, two with cats, three with sheep, one with rabbits, and three with doves and hens. Blood was obtained from anaesthetized animals by cephalic or jugular venipuncture. 

#### 4.2.3. Deer

Serum samples were collected from 30 wild, adult red deer (*Cervus elaphus*) (20 females and 10 males). These were either shot or found dead (victims of road accidents). Blood was obtained from animals by jugular venipuncture. 

The number of different ticks on all sampled animals was recorded following the method of Dominguez-Peñafiel et al. [25].

### 4.3. Immunofluorescence Assays

Sera were examined by IFA as described by Phillip et al. [26]. The rickettsia employed as antigens were *R. slovaca* (strain 246 CDC) and *R. conorii* (strain Malish 7); these were propagated in Vero E6 cells (ATCC CRL 1586) and fixed on spot slides. Appropriate fluorescein-labelled conjugates (rabbits anti-dog IgG, donkey anti-sheep IgG, chicken-anti-cervid; IgG Sigma, St Louis, MO, USA) were used for each animal species. Positive and negative control sera were provided by Dr. Fatima Alves of the Centro de Estudos de Vectores e Doencas Infecciosas, Aguas de Moura, Portugal. Titres equal to or higher than 1/40 were considered positive [27].

### 4.4. Statistical Analysis 

Differences in seroprevalence across different animal species, genders, ages, etc., were examined using the *χ^2^* or Fisher exact test as required. Significance was set at *p* < 0.05. 

## Figures and Tables

**Table 1:** Seroprevalence and antibody titres.

	Titre 1/40		Titre 1/80		Titre 1/160		Titre 1/320		Titre 1/640		Titre 1/1280	
	* RS	** RC	RS	RC	RS	RC	RS	RC	RS	RC	RS	RC
Sheep	5%	4%	5%	4%	0.8%	4%	1.6%	0.8%	-	-	1.6%	-
Dogs	-	1.3%	4%	4%	4%	2.6%	1.3%	-	1.3%	4%	2.6%	1.3%
Deer	13.3%	23.3%	20%	26.6%	16.6%	13.3%	10%	-	10%	3.3%	3.3%	-

* RS: R. slovaca; ** RC: R. conorii.

## Data Availability

Not applicable.
